# Genome Sequence of Eubacterium callanderi AMC0717, Isolated from the Colonic Mucosa of an 11-Year-Old Organ Donor

**DOI:** 10.1128/MRA.00995-20

**Published:** 2020-11-12

**Authors:** Alan J. Marsh, Kshipra Chandrashekhar, Sandy Ng, Jeff Roach, Scott T. Magness, M. Andrea Azcarate-Peril

**Affiliations:** a Department of Medicine, Division of Gastroenterology and Hepatology, School of Medicine, University of North Carolina, Chapel Hill, North Carolina, USA; b UNC Microbiome Core, Center for Gastrointestinal Biology and Disease, School of Medicine, University of North Carolina, Chapel Hill, North Carolina, USA; c UNC Information Technology Services and Research Computing, University of North Carolina, Chapel Hill, North Carolina, USA; d Joint Department of Biomedical Engineering, UNC/NC State University, Chapel Hill, North Carolina, USA; e Department of Cell Biology and Physiology, University of North Carolina, Chapel Hill, North Carolina, USA; University of Maryland School of Medicine

## Abstract

Eubacterium callanderi AMC0717 was isolated from the mucosa of the transverse colon of an 11-year-old organ donor. This strain contains genes putatively encoding short-chain fatty acids (SCFAs), exopolysaccharide (EPS), and several B vitamins.

## ANNOUNCEMENT

Eubacterium callanderi is an anaerobic, non-spore-forming member of the phylum *Firmicutes*, belonging to the true *Eubacterium* cluster within the *Clostridium* XV complex (1). *E. callanderi* has thus far only been isolated from ruminal content and pig feces (2). While *E. callanderi* has been reported as the causative agent in a single case of bacteremia (3), strains are currently listed under biosafety level 1 in the American Type Culture Collection (ATCC) and the German Collection of Microorganisms and Cell Cultures (DSMZ).

Dilutions from the mucosa of the transverse colon of an 11-year-old organ donor were plated onto thioglycolate agar and grown in an anaerobic chamber, after which a pure colony of Eubacterium callanderi AMC0717 was isolated. Notably, unlike some of the strains listed in culture collections, AMC0717 can be cultivated without rumen fluid in prereduced thioglycolate broth. Genomic DNA was isolated (4) and sequenced using the Thermo Fisher Ion GeneStudio S5 system. Raw single-end reads were trimmed and processed using BBDuk tools v38.75 (https://jgi.doe.gov/data-and-tools/bbtools/). A total of 4,401,816 reads were obtained with an average length of 191 bp. Genomes were assembled using SPAdes v3.14.0 (5), assessed for completeness and contamination using CheckM v1.1.2 (6), and annotated using the NCBI Prokaryotic Genome Annotation Pipeline v4.12 (7). Default parameters were used for all software unless otherwise specified.

*E. callanderi* AMC0717 has a genome size of 4,327,770 bp, split across 85 contigs. There are 4,539 coding sequences and 54 RNA genes, with an overall GC content of 47.5%. AMC0717 shares 98.6% average nucleotide identity (ANI) (8) with the type strain, Eubacterium callanderi FD (2), and 93.9% with Eubacterium limosum ATCC 8486, the type species for the genus *Eubacterium*. No plasmids were identified using PlasmidFinder v2.0.1 (9). Phaster (10) identified one intact bacteriophage, a 36.6-kb region most similar to the *Clostridium* phage phiCD111 (11).

**FIG 1:**
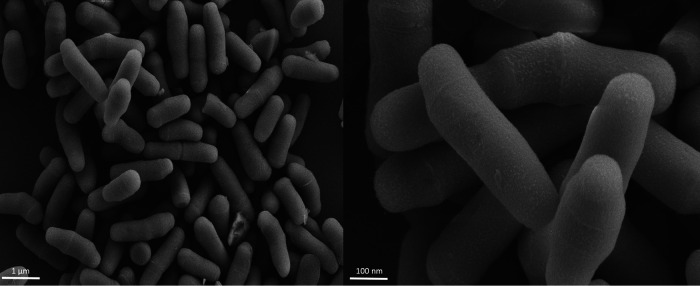
Scanning electron microscope (SEM) imaging of Eubacterium callanderi AMC0717. Briefly, bacterial cell pellets were resuspended in 2% paraformaldehyde/2.5% glutaraldehyde in 0.15 M sodium phosphate buffer, pH 7.4. Following treatment as described previously (17), the fixed cell suspension was deposited onto 12-mm round poly-d-lysine-coated coverslips, which, following preparation, were mounted onto 13-mm aluminum stubs and sputter coated with 5 nm of a gold-palladium alloy. A Zeiss Supra 25 field emission (FE) SEM operating at 5 kV was used to view the AMC0717 isolate with scanning electron microscopy.

CARD v3.0.7 (12) identified a gene with 79% identity with an elfamycin-resistant EF-Tu gene, previously linked to pulvomycin resistance in Escherichia coli. Genes potentially encoding resistance to heavy metals, including mercury, copper, cobalt, zinc, and cadmium, were identified. One gene was identified encoding a toxin of the TlyA family RNA methyltransferase (i.e., a hemolysin). *Eubacterium callanderi* is considered nonmotile; however, a type IV pilus twitching motility protein, PilT (13), was identified in the genome but was not visible when viewed under scanning electron microscopy (Fig. 1). The genome contains a number of genes for exopolysaccharide (EPS) and capsule production, including rhamnose-containing glycans. Putative gene clusters were present for the biosynthesis of three B vitamins, biotin (B7; *bioABDFW*), pyridoxine (B6; *pdxABHFJK*), and folate (B9; *folBEKB*), but further work is required to confirm the functionality of these genes. Homologs to two ribosomally synthesized, posttranslationally modified antimicrobial peptides (sactipeptide and linaridin) were identified using BAGEL4 (14), while gene homologs to terpene and another nonribosomal peptide synthetase were detected with AntiSMASH v5.1 (15). Putative genes belonging to the pyruvate-formate system were found in AMC0717, in addition to homologs for butanol biosynthesis and lactate, acetoin, and butanediol metabolism.

While previously of interest for the production of bioenergy (2, 16), the presence of genes for the synthesis of short-chain fatty acids (SCFAs) and vitamins indicates that AMC0717 may play an important role in gut health.

### Data availability.

This whole-genome shotgun project has been deposited at DDBJ/ENA/GenBank and SRA under the accession numbers JACCKS000000000 and SRR12606937, respectively. Additional information can be found at the AMC Culture Collection (https://redcap.unc.edu/solutions/microbiome_core_986.php).
